# 474. Real-World Experience of the Comparative Effectiveness and Safety of Remdesivir and Combination Therapy (Remdesivir and Monoclonal Antibodies) in Immunosuppressed Patients with Mild to Moderate COVID-19: A Retrospective Single-center Study in a Community Setting

**DOI:** 10.1093/ofid/ofad500.544

**Published:** 2023-11-27

**Authors:** Jun Hirai, Nobuaki Mori, Daisuke Sakanashi, Nobuhiro Asai, Mao Hagihara, Hiroshige Mikamo

**Affiliations:** Aichi Medical University, Nagakute, Aichi, Japan; Aichi Medical University, Nagakute, Aichi, Japan; Aichi Medical University Hospital, Nagakute, Aichi, Japan; Aichi Medical University, Nagakute, Aichi, Japan; Department of Infection Control and Prevention, Aichi Medical University Hospital, Japan., Nagakute, Aichi, Japan; Aichi Medical University, Nagakute, Aichi, Japan

## Abstract

**Background:**

Although several treatment options have been developed for COVID-19, there are still significant challenges in treating certain immunosuppressed patient populations. The objective of this retrospective observational study was to evaluate and compare the effectiveness and safety of immunosuppressed COVID-19 patients treated with remdesivir alone or combination therapy (remdesivir and monoclonal antibodies).

**Methods:**

We included immunosuppression patients who had at least one risk factor for progression to severe COVID-19 from July 2021 to March 2023. The definition of an immunosuppression patient was follows: an autoimmune disease with immunosuppressive therapy, a kidney transplant with an immunosuppressive agent, or having a solid tumor or blood cancer. REGN-COV2 (casirivimab and imdevimab) was prescribed during the Delta strain epidemic season while sotrovimab was prescribed during the Omicron BA1 strain epidemic season. Treatment for each group was initiated within 7 days of symptom onset. We evaluated the time to the resolution of fever over 24 hours after treatment initiation and the Ct value in the study patients.

**Results:**

We enrolled 86 patients who met the inclusion criteria, of whom 35 received combination therapy (CT group) and 51 received remdesivir alone (MT group). No significant difference was observed between the two groups with regard to laboratory data and underlying diseases. There was no difference regarding the duration of remdesivir administration, non-vaccinated population, and number of vaccination between the two groups. A statistically significant reduction in fever of more than 24 hours was achieved in the CT group compared to the MT group (*p* = 0.01). The CT group patients showed a steeper viral load reduction with respect to the MT group (*p* = 0.026). Adverse events such as liver dysfunction and kidney disease occurred in only one patient in each group. On comparing both groups, the duration of hospital stay, the ratio of exacerbation, death by COVID-19, and 30-day all-cause mortality did not differ significantly.

The presentstudy cohort

**Figure:**
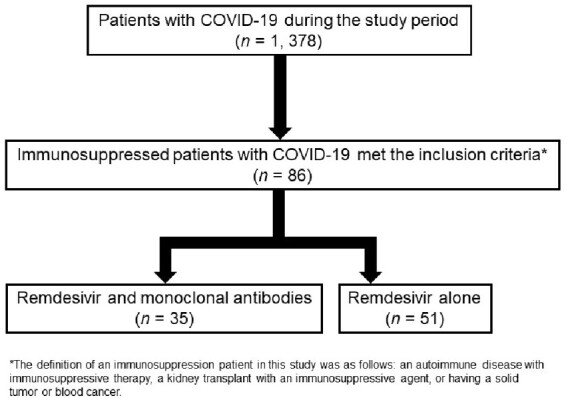

**Figure:**
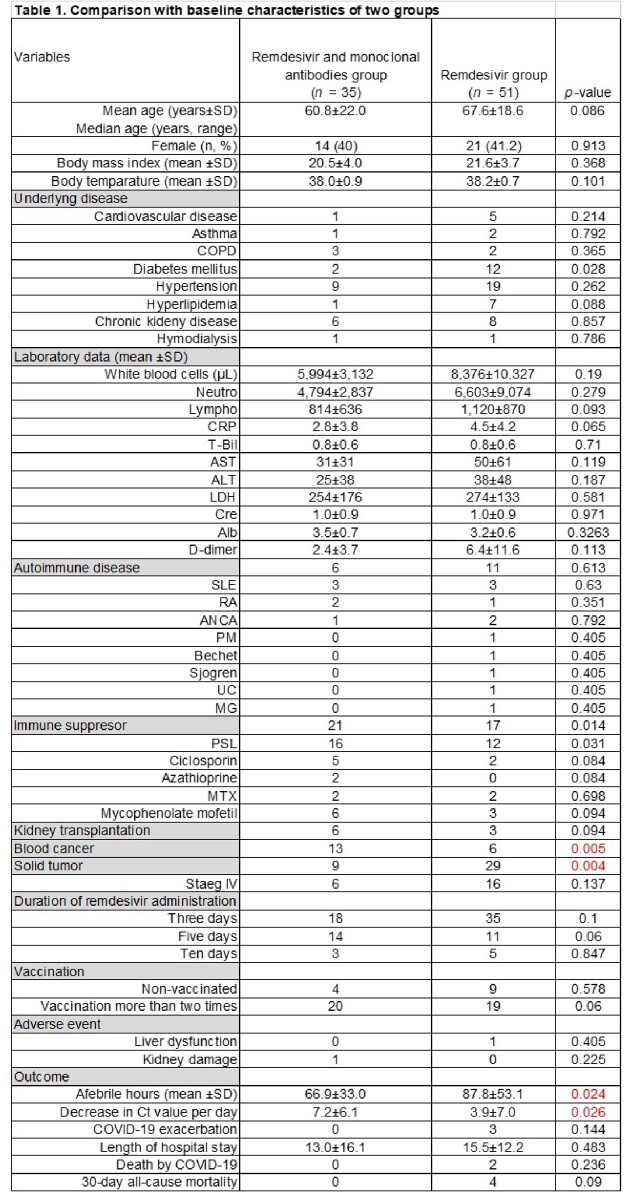

**Conclusion:**

By evaluating the effectiveness of combination therapy with remdesivir and monoclonal antibodies, our study has the potential to improve clinical outcomes and reduce the burden of COVID-19 in this vulnerable population.

**Disclosures:**

**Hiroshige Mikamo, M.D, Ph.D**, Asahi Kasei Pharma Corporation: Grant/Research Support|Merck Sharp & Dohme: Honoraria|Pfizer Inc.: Grant/Research Support|Pfizer R&D Japan: Honoraria|Sumitomo Pharma Co., Ltd.: Grant/Research Support|Sumitomo Pharma Co., Ltd.: Honoraria

